# Strategic white matter hyperintensity locations associated with post-stroke cognitive impairment: A multicenter study in 1568 stroke patients

**DOI:** 10.1177/17474930241252530

**Published:** 2024-06-02

**Authors:** Mirthe Coenen, Floor AS de Kort, Nick A Weaver, Hugo J Kuijf, Hugo P Aben, Hee-Joon Bae, Régis Bordet, Christopher PLH Chen, Anna Dewenter, Thomas Doeven, Thibaut Dondaine, Marco Duering, Rong Fang, Ruben S van der Giessen, Jonguk Kim, Beom Joon Kim, Paul LM de Kort, Peter J Koudstaal, Minwoo Lee, Jae-Sung Lim, Renaud Lopes, Robert J van Oostenbrugge, Julie Staals, Kyung-Ho Yu, Geert Jan Biessels, J Matthijs Biesbroek

**Affiliations:** 1 Department of Neurology and Neurosurgery, UMC Utrecht Brain Center, University Medical Center Utrecht, Utrecht, The Netherlands; 2 Image Sciences Institute, University Medical Center Utrecht, Utrecht, The Netherlands; 3 Department of Neurology, Elisabeth Tweesteden Hospital, Tilburg, The Netherlands; 4 Department of Neurology, Seoul National University Bundang Hospital, Seoul National University College of Medicine, Seongnam, Republic of Korea; 5 Lille Neuroscience & Cognition (LilNCog)—U1172, Université Lille, Inserm, CHU Lille, Lille, France; 6 Department of Pharmacology, Yong Loo Lin School of Medicine, National University of Singapore, Singapore; 7 Memory, Ageing and Cognition Center, National University Health System, Singapore; 8 Institute for Stroke and Dementia Research (ISD), LMU University Hospital, LMU Munich, Munich, Germany; 9 Medical Image Analysis Center (MIAC), Department of Biomedical Engineering, University of Basel, Basel, Switzerland; 10 Department of Neurology, Erasmus Medical Center, Rotterdam, The Netherlands; 11 Department of Neurology, School of Medicine, Inha University, Incheon, Republic of Korea; 12 Department of Neurology, Hallym University Sacred Heart Hospital, Hallym University College of Medicine, Anyang, Republic of Korea; 13 Department of Neurology, Asan Medical Center, University of Ulsan College of Medicine, Seoul, Republic of Korea; 14 Department of Neurology, Maastricht University Medical Center, Maastricht, The Netherlands; 15 Department of Neurology, Diakonessenhuis Hospital, Utrecht, The Netherlands

**Keywords:** White matter hyperintensities, post-stroke cognition, strategic lesion location

## Abstract

**Background:**

Post-stroke cognitive impairment (PSCI) occurs in up to 50% of stroke survivors. Presence of pre-existing vascular brain injury, in particular the extent of white matter hyperintensities (WMH), is associated with worse cognitive outcome after stroke, but the role of WMH location in this association is unclear.

**Aims:**

We determined if WMH in strategic white matter tracts explain cognitive performance after stroke.

**Methods:**

Individual patient data from nine ischemic stroke cohorts with magnetic resonance imaging (MRI) were harmonized through the Meta VCI Map consortium. The association between WMH volumes in strategic tracts and domain-specific cognitive functioning (attention and executive functioning, information processing speed, language and verbal memory) was assessed using linear mixed models and lasso regression. We used a hypothesis-driven design, primarily addressing four white matter tracts known to be strategic in memory clinic patients: the left and right anterior thalamic radiation, forceps major, and left inferior fronto-occipital fasciculus.

**Results:**

The total study sample consisted of 1568 patients (39.9% female, mean age = 67.3 years). Total WMH volume was strongly related to cognitive performance on all four cognitive domains. WMH volume in the left anterior thalamic radiation was significantly associated with cognitive performance on attention and executive functioning and information processing speed and WMH volume in the forceps major with information processing speed. The multivariable lasso regression showed that these associations were independent of age, sex, education, and total infarct volume and had larger coefficients than total WMH volume.

**Conclusion:**

These results show tract-specific relations between WMH volume and cognitive performance after ischemic stroke, independent of total WMH volume. This implies that the concept of strategic lesions in PSCI extends beyond acute infarcts and also involves pre-existing WMH.

**Data access statement:**

The Meta VCI Map consortium is dedicated to data sharing, following our guidelines.

## Introduction

Post-stroke cognitive impairment (PSCI) occurs in up to 50% of stroke survivors.^1,2^ Demographic factors, such as age and education, are known determinants for cognition after stroke. Features of the acute infarct, in particular size and location, are also key determinants of PSCI.^3,4^ In addition, recent evidence points toward an important contribution of pre-existing vascular brain injury to PSCI, for example, white matter hyperintensities (WMH) of presumed vascular origin, lacunes, and old infarcts, as well as brain atrophy.^5^

Most prior studies focused on the relationship of global WMH volume with post-stroke cognition.^6–11^ The question arises whether the concept of strategic lesion locations, that has been established for infarcts, is also relevant for WMH. There is converging evidence in memory clinic patients, patients with cerebral autosomal dominant arteriopathy with subcortical infarcts and leukoencephalopathy (CADASIL) and community-dwelling individuals, that there are indeed several strategic white matter tracts where WMH impact cognition, but this concept has not been sufficiently studied in the setting of stroke.^12,13^ In a previous large multicenter study in memory clinic patients,^14^ separate from this study sample, we identified the left and right anterior thalamic radiation, the forceps major, and the left inferior fronto-occipital fasciculus as strategic white matter tracts, among 20 tracts that were evaluated.

## Aims and hypothesis

The aim of this study is to test the hypothesis that WMH in these four strategic tracts are also inversely associated with cognitive outcome in patients with ischemic stroke, independent of total WMH volume.

## Methods

### Study design and participants

We pooled and harmonized individual patient data from nine ischemic stroke cohorts: France (STROKDEM), Germany (DEDEMAS), the Netherlands (CASPER, CODECS, PROCRAS, USCOG), Singapore (COAST), and South Korea (Bundang VCI, Hallym VCI). Eligible cohorts were derived from two previously published Meta VCI Map projects.^3,15^ Individual patient data from these cohorts were included if magnetic resonance imaging (MRI) with fluid-attenuated inversion recovery (FLAIR) and T1 sequences and detailed neuropsychological assessment within 15 months of the index stroke were available.

Central data processing and analysis were performed at the University Medical Center Utrecht (Utrecht, the Netherlands). For all participating cohorts, ethical and institutional approvals were obtained as required by local regulations, including informed consent, to allow data acquisition and data sharing. Background and organization of the Meta VCI Map consortium are described in the design paper^15^ and on the consortium website (https://metavcimap.org).

### Image processing

For eight cohorts, WMH segmentations were performed in Utrecht as part of the current project, using a semi-automated validated technique.^16,17^ For one cohort, CASPER, WMH segmentations were provided by the participating center. Details on WMH segmentation methods per cohort are provided in the Supplemental Material. WMH lesion maps were registered to the Montreal Neurological Institute (MNI)-152 brain template^18^ for spatial normalization using RegLSM.^19^ Acute infarct maps were subtracted from the WMH lesion map to exclude the possible classification of an infarct as WMH. In addition, voxels located outside the white matter were removed from all individual WMH lesion maps using the MNI probabilistic white matter atlas^20^ (thresholded at 30%). All white matter tracts were defined according to the JHU atlas with a probability threshold of 10%.^21^

Acute infarct segmentations in MNI-152 space were available from previously published Meta VCI Map projects.^3,15^ More details on image processing are described in the Supplemental Material.

### Neuropsychological data

Individual neuropsychological tests were assigned to four cognitive domains: (1) attention and executive functioning, (2) information processing speed, (3) language, and (4) verbal memory. If a cognitive domain was represented by more than one neuropsychological test, the mean of the norm-referenced z-scores from the available neuropsychological tests was obtained. This was done on a per subject basis. Further details on the available neuropsychological tests and harmonization of the neuropsychological data are provided in the Supplemental Material.

### Statistical analyses

In a region of interest (ROI)-based approach, the number of affected voxels with WMH per tract for each patient was related to cognitive performance at the domain level in linear mixed models with cognitive domain z-score as dependent variable, correcting for study site (as random effects) and total WMH volume (as fixed effects). We chose this ROI-based approach because it has the advantage of considering cumulative lesion burden within a single tract (which may be more relevant than considering each punctate lesion separately) and has higher statistical power compared to voxel-based analyses.^14^ Our primary analyses considered four white matter tracts identified as strategic in earlier work^14^ in a memory clinic setting: the left anterior thalamic radiation, right anterior thalamic radiation, forceps major, and the left inferior fronto-occipital fasciculus. In this hypothesis-driven design, we did not correct for multiple comparisons. In secondary analyses, the same models were run, stratified by infarct subtype. For large subcortical and cortical infarcts, we also corrected for infarct volume (as fixed effects). A p-value of <0.05 was considered as statistically significant. For all ROI-based analyses, patients with an acute infarct in one of the white matter tracts were excluded from the analysis of that specific white matter tract.

Second, a sensitivity analysis was performed including all 20 white matter tracts from the JHU atlas, in order to assess whether relevant white matter tracts were missed in the primary analysis. As this analysis was not hypothesis-driven, a Bonferroni correction for 20 tests (i.e. 20 ROIs for major white matter tracts) was applied, and a p-value < 0.0025 was considered statistically significant.

Third, a multivariable lasso regression model (using 100-fold cross-validations) was used to study the significant findings from the primary analysis in more detail, including WMH volume in strategic WMH tracts and total WMH volume, total infarct volume, as well as age, sex, and education as covariates. The lasso regression allows to assess the individual contribution of the white matter tracts in a multivariable model, thereby correcting for other determinants of post-stroke cognition (i.e. WMH volume and infarct volume), also considering multicollinearity between the independent variables (i.e. total WMH volume, total infarct volume, the strategic WMH tracts). The model shrinks the regression coefficients down to zero if the variable is redundant. The independent continuous variables were standardized and the model was corrected for study site. In this way, the individual contribution of each independent variable to the cognitive domain scores could be determined.

All ROI-based analyses were performed using glmnet (v4.1.3) and lme4 (v1.1.26) in R (v4.1.2).

## Results

### Participants

**Figure 1.**
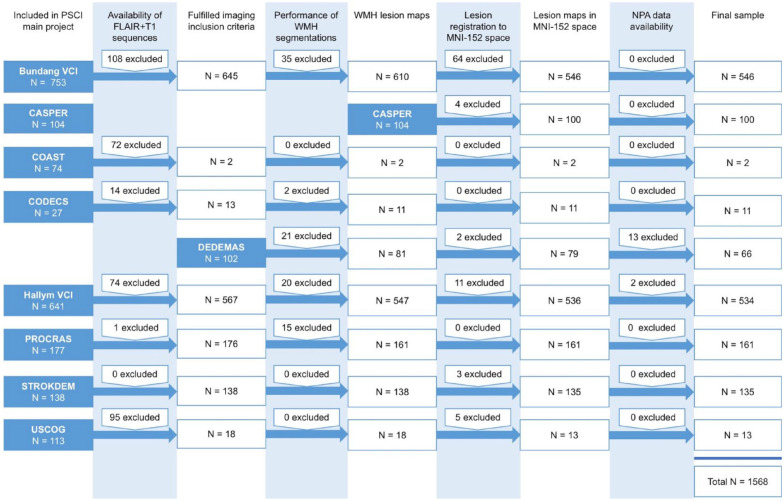
Flowchart of patient selection. This figure shows the flowchart of patient selection for the nine participating cohorts. This figure was previously published in Stroke.^11^

**Figure 2.**
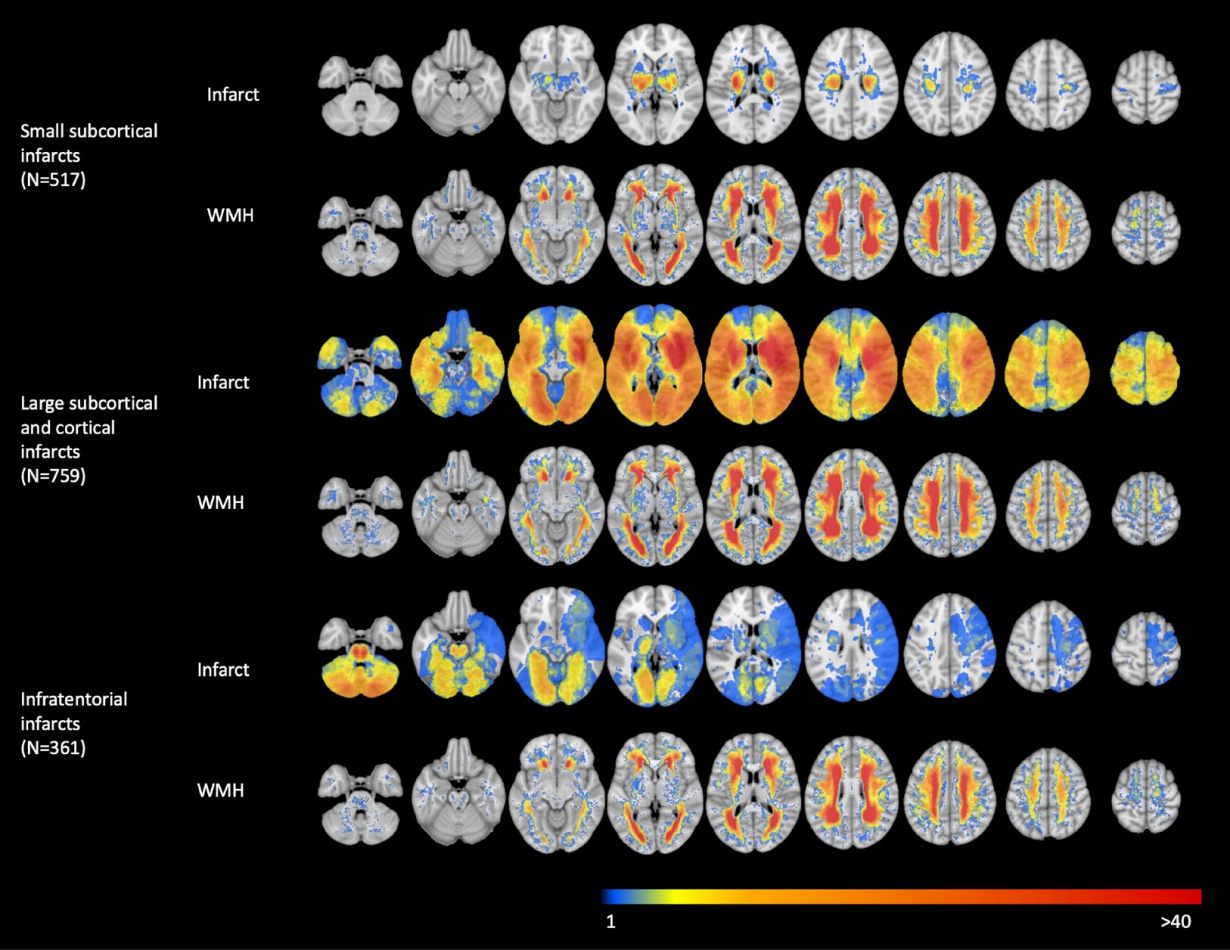
Infarct and WMH prevalence maps shown per infarct subtype. This figure shows how often each location in the brain was affected by an acute infarct or WMH. Blue voxels are damaged in one patient and red voxels in >40 patients. Results are presented per infarct subtype (i.e. small subcortical infarcts, large subcortical and cortical infarcts, and infratentorial infarcts). For each infarct subtype, the first row represents the infarct prevalence map and the second row represents the WMH prevalence map. Patients with multiple infarcts can be represented in more than one stroke category.

A flowchart of patient selection from the participating cohorts is provided in Figure 1. The total study sample consisted of 1568 patients (39.9% female) from 9 stroke cohorts, with a mean age of 67.3 years (standard deviation (SD) = 11.5). In the total study sample, the mean norm-referenced cognitive domain z-score for attention and executive functioning was −0.7 (SD = 1.0), information processing speed −0.6 (SD = 1.2), language −0.7 (SD = 1.1), and verbal memory −0.9 (SD = 1.2). Of total, 517 (33.0%) patients had a small subcortical infarct, 759 (48.4%) patients had a large subcortical or cortical infarct, and 361 (23.0%) patients had an infratentorial infarct. Median WMH volume was 7.1 mL (interquartile range (IQR) = 16.4). Baseline characteristics of the total study sample and per infarct subtype are shown in Table 1 and cohort-specific baseline characteristics in Supplemental Table S2. Lesion prevalence maps of the acute infarct and WMH per infarct subtype are shown in Figure 2.

**Table 1.** Baseline characteristics.

Characteristics	Infarct subtype			Total (n = 1568)
	Small subcortical infarct (n = 517)	Large subcortical and cortical infarct (n = 759)	Infratentorial infarct (n = 361)	
Female, n (%)	235 (45.5)^*^	290 (38.2)^*^	121 (33.5)^*^	626 (39.9)^*^
Age in years, mean (SD)	66.3 (11.7)^*^	67.7 (11.7)^*^	68.1 (11.4)^*^	67.3 (11.5)^*^
Education, n (%)				
Lower than secondary school	265 (51.3)^*^	398 (52.4)^*^	188 (52.1)^*^	816 (52.0)^*^
Secondary school	122 (23.6)^*^	164 (21.6)^*^	71 (19.7)^*^	343 (21.9)^*^
Technical school or college	57 (11.0)^*^	65 (8.6)^*^	33 (9.1)^*^	153 (9.8)^*^
University or higher	73 (14.1)^*^	132 (17.4)^*^	69 (19.1)^*^	256 (16.3)^*^
Baseline NIHSS, mean (SD)	2.5 (2.5)^#^	4.3 (4.9)^#^	3.1 (3.6)^#^	3.5 (4.0)^#^
History of stroke and TIA, n (%)	64 (12.9)^#^	114 (15.0)^#^	51 (14.1)^#^	217 (14.4)^#^
Imaging characteristics				
Normalized acute infarct volume (mL), median (IQR)	1.1 (1.2)^*^	11.2 (22.2)^*^	1.7 (6.7)^*^	2.6 (11.4)^*^
Presence of an old infarct, n (%)	73 (14.1)	177 (23.3)	73 (20.2)	302 (19.3)
Normalized WMH volume (mL), median (IQR)	8.3 (17.0)^*^	6.0 (15.4)^*^	8.6 (18.7)^*^	7.1 (16.4)^*^
Cognitive performance, mean (SD)				
MoCA	23.5 (4.0)^*^	22.3 (4.6)^$^	23.5 (9.4)^$^	23.0 (4.4)^$^
Domain z-score				
Attention and executive functioning (n = 1470)	−0.6 (1.0)^#^	−0.9 (1.1)^#^	−0.6 (1.0)^#^	−0.7 (1.0)^#^
Information processing speed (n = 1467)	−0.5 (1.1)^#^	−0.8 (1.2)^#^	−0.5 (1.0)^#^	−0.6 (1.2)^#^
Language (n = 1537)	−0.6 (1.0)^#^	−0.9 (1.1)^#^	−0.7 (1.0)^#^	−0.7 (1.1)^#^
Verbal memory (n = 1545)	−0.8 (1.2)^#^	−1.1 (1.3)^#^	−0.9 (1.1)^#^	−0.9 (1.2)^#^

SD: standard deviation; NIHSS: National Institutes of Health Stroke Scale; TIA: transient ischemic attack; IQR: interquartile range; WMH: white matter hyperintensity; MoCA: Montreal Cognitive Assessment.

Patients with multiple infarcts can be represented in more than one stroke category.

* Missing in < 1%.

# Missing in 1–10%.

$ Missing in > 10%.

### WMH tract–based linear mixed models

In the primary analysis (see Table 2) including four preselected white matter tracts, a significant negative correlation was found between WMH volume within the left anterior thalamic radiation and attention and executive functioning (coefficient = −0.175, standard error (SE) = 0.065, p = 0.007) and information processing speed (coefficient = −0.197, SE = 0.071, p = 0.006) and between WMH volume within the forceps major and information processing speed (coefficient = −0.132, SE = 0.054, p = 0.014), independent of total WMH volume and study site. No significant correlations were found between the right anterior thalamic radiation and left inferior fronto-occipital fasciculus and cognitive functioning.

**Table 2.** Results from the linear mixed model analysis including the four preselected white matter tracts.

Model	Independent variables	Attention and executive functioning			Information processing speed			Language			Verbal memory		
		Coefficient	SE	p-value	Coefficient	SE	p-value	Coefficient	SE	p-value	Coefficient	SE	p-value
1	WMH volume	−0.010	0.001	< 0.001*	−0.011	0.002	< 0.001*	−0.009	0.001	< 0.001*	−0.003	0.001	0.017*
2	Model 1 + anterior thalamic radiation L	−0.175	0.065	0.007*	−0.197	0.071	0.006*	−0.078	0.058	0.175	−0.089	0.062	0.154
3	Model 1 + anterior thalamic radiation R	−0.089	0.071	0.212	−0.117	0.077	0.13	−0.096	0.067	0.149	−0.076	0.071	0.282
4	Model 1 + forceps major	−0.076	0.051	0.131	−0.132	0.054	0.014*	0.010	0.046	0.837	−0.049	0.050	0.327
5	Model 1 + inferior fronto-occipital fasciculus L	−0.088	0.080	0.271	0.010	0.088	0.900	0.069	0.069	0.321	−0.039	0.077	0.606

SE: standard error; WMH: white matter hyperintensity.

This table shows the results of the ROI-based analysis using a linear mixed model including four preselected white matter tracts. Patients with an infarct in the white matter tract of interest were excluded from the analysis. The results are corrected for study site using random effects. A p-value < 0.05 was considered statistically significant (indicated by *). The independent variables (i.e. total WMH volume and regional WMH volumes) are not standardized. The coefficient therefore corresponds to the change in cognitive functioning (decrease or increase in z-score) associated with each 1 mL increase of the independent variables.

In the sensitivity analyses, considering all 20 white matter tracts defined according to the JHU atlas, one additional significant inverse association was found, namely between WMH volume within the right inferior longitudinal fasciculus and verbal memory (Supplemental Table S3).

Stratification of the main analysis according to infarct subtype showed consistent results with the overall analyses for cortical and large subcortical infarcts and small subcortical infarcts, whereas for infratentorial infarcts, the relations between tract-specific WMH volume and cognition were attenuated (Supplemental Table S4).

### Multivariable lasso regression models

Three multivariable lasso regression models were performed on the significant results from the primary analysis. In these models (including age, sex, education, total infarct volume, total WMH volume, tract specific WMH volume, and study site as independent variables), WMH volume within the left anterior thalamic radiation had a larger coefficient when assessing attention and executive functioning (−0.164) and information processing speed (−0.121) than total WMH volume (−0.012 and −0.073, respectively) and WMH volume within the forceps major had a larger coefficient when assessing information processing speed (−0.126) than total WMH volume (−0.090) (Table 3).

**Table 3.** Lasso regression results.

Model	Independent variables	Attention and executive functioning	Information processing speed
		Coefficient	Coefficient
1	Age	−0.007	−0.083
	Sex	R	R
	Education	R	R
	Total infarct volume	−0.101	−0.205
	Total WMH volume	−0.012	−0.073
	WMH volume anterior thalamic radiation L	−0.164	−0.121
2	Age		−0.020
	Sex		R
	Education		0.082
	Total infarct volume		−0.138
	Total WMH volume		−0.090
	WMH volume forceps major		−0.126

WMH: white matter hyperintensity

A lasso regression model was used to relate total infarct volume, total WMH volume, tract-specific WMH volume (model 1: left anterior thalamic radiation, model 2: Forceps major), as well as age, sex (0: female, 1: male), and education to each of the four cognitive domains. Lasso regression corrects for multicollinearity between independent variables and reduces the coefficients to zero (indicated by R in the table) if the variable is redundant. Apart from sex, all independent variables are standardized, which means the reported coefficients correspond with the change in cognitive functioning (i.e. increase or decrease in z-score) associated with 1 SD change in the independent variable. The model is also corrected for the nine study sites by including these as dummy variables (coefficients of study sites are not shown).

## Discussion

In this large multicenter study, we found that WMH in strategic white matter tracts was associated with domain-specific cognitive functioning in patients with ischemic stroke. Tract-based analyses showed that regional WMH volume within the left anterior thalamic radiation and the forceps major inversely correlated with attention and executive functioning and information processing speed. The effect sizes of individual white matter tracts—reflected in the coefficients of lasso regression—were larger than for total WMH volume and the tract-specific relations with cognition were independent of infarct volume, age, sex, and level of education. Stratification of the results according to infarct subtype showed that these effects were consistent in patients with large subcortical, cortical, and small subcortical infarcts and attenuated in patients with infratentorial infarcts.

Previous studies on WMH location in other clinical settings, that is memory clinic patients (including vascular and/or Alzheimer disease), for example, Coenen et al.,^14^ CADASIL,^22,23^ and community-dwelling individuals, for example, Lampe et al.^24^ and Biesbroek et al.^25^ already generated evidence that the cognitive impact of WMH depends on location. The anterior thalamic radiation and corpus callosum have been identified most consistently in these studies.^14,26,27^ The anterior thalamic radiation connects the anterior and dorsomedial thalamic nuclei with the frontal and cingulate cortices. The forceps major connects the occipital lobes through the splenium of the corpus callosum, which forms the main connection between the cerebral hemispheres.^28^ Thus, these tracts form part of large distributed brain networks, which adds plausibility to the finding that WMH in these tracts are associated with executive functioning and processing speed. In a previous large multicenter study including 3525 memory clinic patients,^14^ we identified four specific white matter tracts as strategic white matter tracts which were a determinant of domain-specific cognitive functioning. These four tracts were also included in this study.

For several reasons, it is important to address the question of strategic WMH locations also specifically in the context of ischemic stroke. First, the etiology and distribution of WMH may differ according to disease setting and that may affect the relation with cognition. For example, particularly in memory clinic populations, common occurrence of amyloid pathology may affect WMH distributions.^29^ Moreover, co-occurring pathologies differ in different disease settings and the impact of the acute infarct itself, for example, could in theory overshadow effects of WMH location. So far, only two previous studies (with relatively small sample sizes of 76^12^ and 118^13^ patients) investigated the concept of strategic WMH location for cognition after ischemic stroke. Zhao et al.^12^ found clusters of significant WMH voxels in the corpus callosum, corona radiata, and posterior thalamic radiation. The study from Valdés Hernández et al.^13^ found that an increase of WMH from baseline to 1-year follow-up in the external capsule, intersection between the anterior limb of the internal and external capsules, and optical radiation, was associated with worse post-stroke cognitive functioning. Direct comparison between our results and these prior studies is complicated by the use of different anatomical parcellations and terminology for reporting strategic regions across studies, but based on a visual inspection of these prior results, the left anterior thalamic radiation and forceps major appear to be consistently identified as strategic white matter tracts.

Strengths of this study are the large sample size. Inclusion of individual patient data from nine cohorts from multiple continents provides geographical and ethnical diversity. Due to the large sample size, we were also able to stratify for infarct subtype, an important aspect given that different infarct subtypes are differentially related to WMH and stroke outcomes. Second, using a previously validated image processing pipeline,^19^ we were able to harmonize data from different centers using different scanners and MRI acquisition protocols. Third, the availability of detailed neuropsychological data made it possible to assess post-stroke cognitive functioning on a continuous outcome scale on the level of four different cognitive domains. Fourth, we obtained a high statistical power of the primary analysis using a hypothesis-driven approach including four preselected white matter tracts. However, this approach assumes generalizability of the strategic white matter tracts from a memory clinic setting to patients with ischemic stroke. Therefore, we also performed sensitivity analyses, which demonstrated that we did not miss any tracts with particular relevance in the context of stroke. Several other limitations also need to be considered. Although patients with pre-stroke dementia were excluded by most participating cohorts, based on either the Informant Questionnaire for Cognitive Decline in the Elderly score or prehospital diagnosis, PSCI could have influenced our findings.^3^ Unfortunately, we were not able to correct for global brain atrophy or medial temporal lobe atrophy in our analyses, because due to technical restraints, brain atrophy measures could not be obtained for a sufficient number of patients. It should also be noted that using a tract-based approach, we only included the 40% of the white matter which is considered in the JHU atlas. Also, the JHU atlas was derived using a simple tractography approach that largely neglects crossing fibers and thus potentially limits anatomical precision. For all patients, the acute infarct maps were subtracted from the WMH maps which might have led to an underestimation of total WMH volume, especially in patients with cortical and large subcortical infarcts. In addition, it was not possible to correct for infarcts in specific white matter tracts; therefore, patients with infarcts in a specific white matter tract were excluded from analyses considering that specific tract. Finally, WMH is a less sensitive marker of white matter injury compared to, for example, Diffusion Tensor Imaging (DTI).^30^ Hence, future DTI studies might provide additional insights into strategic tracts for PSCI.

Implications of this study are that strategic WMH locations should be taken into account to optimally assess the effect of WMH on cognition after stroke at the level of an individual patient. Future studies could use these findings to develop multimodal prediction models that also include other determinants of post-stroke cognition, such as age, sex, and education, together with information on multiple lesion types and their locations, including the acute infarct, prior infarcts, and WMH.

In summary, we identified the left anterior thalamic radiation and the forceps major as strategic white matter tracts related to domain-specific cognitive functioning in stroke patients. These results are in line with current pathophysiological models of PSCI that include pre-existing brain resilience and lesion burden as important determinants that may either protect against or predispose to cognitive impairment after stroke. As such, pre-existing strategic WMH may be relevant in explaining variation in cognitive outcomes after stroke, beyond what is already explained by total WMH volume and clinical and acute infarct characteristics.

## Supplemental Material

sj-docx-1-wso-10.1177_17474930241252530 – Supplemental material for Strategic white matter hyperintensity locations associated with post-stroke cognitive impairment: A multicenter study in 1568 stroke patients Supplemental material, sj-docx-1-wso-10.1177_17474930241252530 for Strategic white matter hyperintensity locations associated with post-stroke cognitive impairment: A multicenter study in 1568 stroke patients by Mirthe Coenen, Floor AS de Kort, Nick A Weaver, Hugo J Kuijf, Hugo P Aben, Hee-Joon Bae, Régis Bordet, Christopher PLH Chen, Anna Dewenter, Thomas Doeven, Thibaut Dondaine, Marco Duering, Rong Fang, Ruben S van der Giessen, Jonguk Kim, Beom Joon Kim, Paul LM de Kort, Peter J Koudstaal, Minwoo Lee, Jae-Sung Lim, Renaud Lopes, Robert J van Oostenbrugge, Julie Staals, Kyung-Ho Yu, Geert Jan Biessels and J Matthijs Biesbroek in International Journal of Stroke

## References

[1] Pendlebury ST Rothwell PM. Incidence and prevalence of dementia associated with transient ischaemic attack and stroke: analysis of the population-based Oxford Vascular Study. Lancet Neurol 2019; 18: 248–258. 30784556 10.1016/S1474-4422(18)30442-3 PMC6390174

[2] Barbay M Taillia H Nédélec-Ciceri C , et al. Prevalence of poststroke neurocognitive disorders using National Institute of Neurological Disorders and Stroke-Canadian Stroke Network, VASCOG criteria (Vascular Behavioral and Cognitive Disorders), and optimized criteria of cognitive deficit. Stroke 2018; 49: 1141–1147. 29643258 10.1161/STROKEAHA.117.018889

[3] Weaver NA Kuijf HJ Aben HP , et al. Strategic infarct locations for post-stroke cognitive impairment: a pooled analysis of individual patient data from 12 acute ischaemic stroke cohorts. Lancet Neurol 2021; 20: 448–459. 33901427 10.1016/S1474-4422(21)00060-0

[4] Zhao L Biesbroek JM Shi L , et al. Strategic infarct location for post-stroke cognitive impairment: a multivariate lesion-symptom mapping study. J Cereb Blood Flow Metab 2018; 38: 1299–1311. 28895445 10.1177/0271678X17728162 PMC6092771

[5] Georgakis MK Duering M Wardlaw JM , et al. WMH and long-term outcomes in ischemic stroke: a systematic review and meta-analysis. Neurology 2019; 92: e1298–e1308. 10.1212/WNL.0000000000007142 30770431

[6] Burton EJ Kenny RA O’Brien J , et al. White matter hyperintensities are associated with impairment of memory, attention, and global cognitive performance in older stroke patients. Stroke 2004; 35: 1270–1275. 15118186 10.1161/01.STR.0000126041.99024.86

[7] Ihle-Hansen H Thommessen B Fagerland MW , et al. Impact of white matter lesions on cognition in stroke patients free from pre-stroke cognitive impairment: a one-year follow-up study. Dement Geriatr Cogn Dis Extra 2012; 2: 38–47. 22619660 10.1159/000336817 PMC3350344

[8] Wright A Tippett D Saxena S , et al. Leukoaraiosis is independently associated with naming outcome in poststroke aphasia. Neurology 2018; 91: e526–e532. 10.1212/WNL.0000000000005945 PMC6105047 29980639

[9] Sachdev PS Brodaty H Valenzuela MJ , et al. The neuropsychological profile of vascular cognitive impairment in stroke and TIA patients. Neurology 2004; 62: 912–919. 15037692 10.1212/01.wnl.0000115108.65264.4b

[10] Jokinen H Kalska H Mäntylä R , et al. White matter hyperintensities as a predictor of neuropsychological deficits post-stroke. J Neurol Neurosurg Psychiatry 2005; 76: 1229–1233. 16107356 10.1136/jnnp.2004.055657 PMC1739804

[11] De Kort FAS Coenen M Weaver NA , et al. White matter hyperintensity volume and poststroke cognition: an individual patient data pooled analysis of 9 ischemic stroke cohort studies. Stroke 2023; 54: 3021–3029. 37901947 10.1161/STROKEAHA.123.044297 PMC10664782

[12] Zhao L Wong A Luo Y , et al. The additional contribution of white matter hyperintensity location to post-stroke cognitive impairment: insights from a multiple-lesion symptom mapping study. Front Neurosci 2018; 12: 290. 29765301 10.3389/fnins.2018.00290 PMC5938410

[13] Valdés Hernández MDC Grimsley-Moore T Chappell FM , et al. Post-stroke cognition at 1 and 3 years is influenced by the location of white matter hyperintensities in patients with lacunar stroke. Front Neurol 2021; 12: 634460. 33732208 10.3389/fneur.2021.634460 PMC7956970

[14] Coenen M Kuijf HJ Huenges Wajer IMC , et al. Strategic white matter hyperintensity locations for cognitive impairment: a multicenter lesion–symptom mapping study in 3525 memory clinic patients. Alzheimers Dement 2023; 19: 2420–2432. 36504357 10.1002/alz.12827

[15] Weaver NA Zhao L Biesbroek JM , et al. The Meta VCI Map consortium for meta-analyses on strategic lesion locations for vascular cognitive impairment using lesion-symptom mapping: design and multicenter pilot study. Alzheimers Dement 2019; 11: 310–326. 10.1016/j.dadm.2019.02.007 PMC6465616 31011619

[16] Kuijf HJ Biesbroek JM De Bresser J , et al. Standardized assessment of automatic segmentation of white matter hyperintensities and results of the WMH segmentation challenge. IEEE Trans Med Imaging 2019; 38: 2556–2568. 30908194 10.1109/TMI.2019.2905770 PMC7590957

[17] Camarasa R Doué C De Bruijne M Dubost F. Segmentation of white matter hyperintensities with an ensemble of multi-dimensional convolutional gated recurrent units, https://wmh.isi.uu.nl/wp-content/uploads/2018/08/coroflo.pdf (2018, accessed 7 November 2022).

[18] Fonov V Evans AC Botteron K , et al. Unbiased average age-appropriate atlases for pediatric studies. Neuroimage 2011; 54: 313–327. 20656036 10.1016/j.neuroimage.2010.07.033 PMC2962759

[19] Biesbroek JM Kuijf HJ Weaver NA , et al. Brain infarct segmentation and registration on MRI or CT for lesion-symptom mapping. J Vis Exp. Epub ahead of print 25 September 2019. DOI: 10.3791/59653. 31609325

[20] Fonov V Evans A McKinstry R Almli CR Collins DL. Unbiased nonlinear average age-appropriate brain templates from birth to adulthood. Neuroimage 2009; 47: S102.

[21] Hua K Zhang J Wakana S , et al. Tract probability maps in stereotaxic spaces: analyses of white matter anatomy and tract-specific quantification. Neuroimage 2008; 39: 336–347. 17931890 10.1016/j.neuroimage.2007.07.053 PMC2724595

[22] Duering M Gonik M Malik R , et al. Identification of a strategic brain network underlying processing speed deficits in vascular cognitive impairment. Neuroimage 2013; 66: 177–183. 23153965 10.1016/j.neuroimage.2012.10.084

[23] Duering M Zieren N Hervé D , et al. Strategic role of frontal white matter tracts in vascular cognitive impairment: a voxel-based lesion-symptom mapping study in CADASIL. Brain 2011; 134: 2366–2375. 21764819 10.1093/brain/awr169

[24] Lampe L Kharabian-Masouleh S Kynast J , et al. Lesion location matters: the relationships between white matter hyperintensities on cognition in the healthy elderly. J Cereb Blood Flow Metab 2019; 39: 36–43. 29106319 10.1177/0271678X17740501 PMC6311671

[25] Biesbroek JM Kuijf HJ Van der Graaf Y , et al. Association between subcortical vascular lesion location and cognition: a voxel-based and tract-based lesion-symptom mapping study. The SMART-MR study. PLoS One 2013; 8: e60541. 10.1371/journal.pone.0060541 PMC3620525 23593238

[26] Biesbroek JM Weaver NA Biessels GJ. Lesion location and cognitive impact of cerebral small vessel disease. Clin Sci 2017; 131: 715–728. 10.1042/CS20160452 28385827

[27] Smith EE Salat DH Jeng J , et al. Correlations between MRI white matter lesion location and executive function and episodic memory. Neurology 2011; 76: 1492–1499. 21518999 10.1212/WNL.0b013e318217e7c8 PMC3087468

[28] Kahle W Frotscher M. Color atlas and textbook of human anatomy: nervous systems and sensory organs. 5th ed. New York: Georg Thieme, 2003.

[29] Weaver NA Doeven T Barkhof F , et al. Cerebral amyloid burden is associated with white matter hyperintensity location in specific posterior white matter regions. Neurobiol Aging 2019; 84: 225–234. 31500909 10.1016/j.neurobiolaging.2019.08.001

[30] Baykara E Gesierich B Adam R , et al. A novel imaging marker for small vessel disease based on skeletonization of white matter tracts and diffusion histograms. Ann Neurol 2016; 80: 581–592. 27518166 10.1002/ana.24758

