# Supplementary material for: Strategic white matter hyperintensity locations associated with post-stroke cognitive impairment: A multicenter study in 1568 stroke patients
Source: Int J Stroke. 2024 Jun 2;19(8):916–24. doi: 10.1177/17474930241252530 (PMC11408955; doi:10.1177/17474930241252530)
Supplement: sj-docx-1-wso-10.1177_17474930241252530 – Supplemental material for Strategic white matter hyperintensity locations associated with post-stroke cognitive impairment: A multicenter study in 1568 stroke patients [file sj-docx-1-wso-10.1177_17474930241252530.docx]

**Supplementary material**

Index

Image processing p.2
Neuropsychological data p.4
Disclosures related to the original cohort studies p.6

Acknowledgments p.7

Supplemental table S1: selection of cognitive tests per cohort p.8

Supplemental table S2: cohort-specific baseline characteristics p.10

Supplemental table S3: sensitivity analyses p.13

Supplemental table S4: stratified analyses p.19

Supplemental references p.24

Image processing^1^

*WMH segmentations*For eight cohorts WMH segmentations were performed in Utrecht as part of Meta VCI Map projects, using open access fully automated techniques.^2^ The best segmentation method was selected on a per cohort basis after visual inspection of the segmentation results. For six cohorts (i.e. Bundang VCI, CODECS, DEDEMAS, Hallym VCI, PROCRAS, and STROKDEM) the coroflo segmentation method was selected^3^ whereas for COAST the bigrbrain segmentation method was selected^4^. For USCOG it was not possible to select a segmentation method on a per cohort basis due to heterogeneity of the imaging data and differences in quality of the different segmentation methods per patient. Therefore, selection of the segmentation method for USCOG was performed on a per subject basis. An expert (MC) with extensive experience on WMH segmentations visually inspected all segmentations. Scans with major disturbances due to technical issues such as scan quality or movement artefacts, or due to old infarcts or other pathology with apparent impact on WMH segmentations and thus volume estimates of an individual patient, were excluded. The acute infarct segmentations were subtracted from the WMH maps at a subsequent processing step. In total 93 (5.3%) of all segmentations failed and were excluded. For CASPER WMH segmentations were provided by the participating center, details are described elsewhere^5^.

*Registration to the MNI-152 template*

The registration of WMH segmentations to the MNI-152 brain template was performed centrally using RegLSM^6^. The FLAIR images were first registered to the corresponding T1 image with a linear registration. The T1 image was subsequently transformed to the T1 1-mm MNI-152 template, with a linear registration followed by a non-linear registration. An age-specific MRI template was used as an intermediate step before the final registration to MNI-152 space in order to improve the quality of the registration by providing a better match between patient and template^7^. The resulting transformations were combined into a single transformation that was subsequently used to transform the corresponding WMH map to the MNI-152 template. The final registration results of all cases were visually checked for accuracy and 89 patients (5.3%) with failed registrations were excluded. To reduce heterogeneity and minimize the effects of possible misclassifications of other lesion types as WMH during the WMH segmentation procedures, voxels located outside the white matter (defined using the MNI probabilistic white matter atlas thresholded^7^ at 30%) were removed from all individual WMH maps. As a final processing step, lesion maps of the acute infarct were subtracted from the WMH maps.

*Definition of infarct subtypes*

Four infarct subtypes were defined: (A) Small subcortical infarcts: single supratentorial infarct without cortical involvement, with a lesion volume of ≤4.19 ml (i.e. a sphere of ≤2 cm diameter; following the STRIVE criteria^8^). (B) Large subcortical infarcts: supratentorial infarct(s) without cortical involvement, with a lesion volume of >4.19 ml. (C) Cortical infarcts: supratentorial infarct(s) of any volume with cortical involvement. (D) Infratentorial infarcts: any brain stem and/or cerebellar infarct(s). Details are described in Weaver et al. (2021)^9^.

For subgroup analyses, group B (large subcortical infarcts) and group C (cortical infarcts) were combined, henceforth referred to as “large subcortical and cortical infarcts”.

Patients who had both an infratentorial infarct and cortical or large subcortical infarct were included in two categories (n=69).

*Global atrophy*

Whole brain segmentations (which were used to calculate the brain parenchymal fraction, a substitute for global atrophy) failed in a substantial number of patients and were therefore only available for 27% of patients.

Neuropsychological data^1^

*Selection of neuropsychological tests*
To reliably compare individual performance (z-scores) on cognitive domains, heterogeneity between cohorts was minimized by only selecting neuropsychological tests that were available in at least 40% of cohorts (n≥4) (selected tests were either truly identical or equivalent in both difficulty and cognitive construct measured). This process resulted in the selection of the following neuropsychological tests: TMT B, Digit Span Forward, Digit Span Backward, Phonemic Fluency (both 2 and 3 letter tests), Semantic Fluency (animal naming), TMT A, WAIS-R Digit Symbol Substitution Test (equivalent: Symbol Digit Modalities test) and the Boston Naming Test (equivalent: French D080 picture naming test). For verbal memory we included all word list recall tests measuring at least two of the following constructs: immediate recall, delayed recall and recognition (Rey Auditory Verbal Learning Test, Seoul Verbal Learning test, Word-List Recall, Free and Cued Selective Reminding Test and, the Word List Memory Task). Allocation of tests to specific cognitive domains was based on previous work^9^. Table S1 shows the selection of neuropsychological tests for each cohort.

*Norm-referenced data*
Cognitive performance at the level of individual neuropsychological tests was determined using local norms or normative data (corrected for age, educational level and sex where appropriate). Z-scores were calculated by the Utrecht team for the CODECS and USCOG cohorts, all other cohorts provided norm-referenced percentile scores or z-scores for each individual test. Percentile scores were converted to z-scores accordingly. Normative data for neuropsychological assessment per cohort are described in the supplements of prior work^9^. In addition, for DEDEMAS, the following norm-data were used: (1) z-scores of CERAD test battery^10^ (including TMT B, Phonemic fluency, TMT A, Boston Naming Test, Semantic fluency-animals, Word-List Memory Task) were based on published norms using a standardized program (2) Z-scores of Digit Symbol Coding were calculated based on normative scores of Wechsler Adult Intelligence Scale, Third Edition (WAIS-III)^11^.

*Identification of outliers and construction of cognitive domain z-scores*Extreme scores were defined as the mean z-score of an individual test +/-3SD, on a per cohort basis. Extreme scores differed per test and per cohort and are probably the result of a combination of specific test characteristics (i.e. time-related tasks are more prone to generating extreme scores), patient characteristics (i.e. floor effects) and norm characteristics, that all differ per cohort. To reduce the impact of these (likely exaggerated) extreme z-scores, all extreme scores were set back to the cut-off value of the mean of the individual test +/-3SD for each individual cohort. The final z-scores of individual tests were used to calculate cognitive domain z-scores (mean of all available z-scores within one domain).

Disclosures related to the original cohort studies

H-JB reports grants from Astrazeneca, Bayer Korea, Bristol Myers Squibb Korea, Chong Gun Dang Pharmaceutical Corp., Dong-A ST, Jeil Pharmaceutical Co., Ltd., Korean Drug Co., Ltd., Samjin Pharm, Takeda Pharmaceuticals Korea Co., Ltd., and Yuhan Corporation, roles as a principal investigator or co-investigator of clinical trials sponsored by Bayer, Bristol Myers Squibb, Dong-A ST, GNT Pharma, Korean Drug Co., Ltd., SAMJIN Pharm, Shinpoong Pharm. Co., Ltd., and personal fees from Amgen Korea, Bayer, Daiichi Sankyo, JW Pharmaceutical, Hanmi Pharmaceutical Co., Ltd., Otsuka Korea, SK chemicals, and Viatris Korea, outside the submitted work. All other authors declare no competing interests.

Acknowledgments

We thank Lei Zhao for his contribution to imaging data harmonization of the Meta VCI Map project data. We thank Guido Cammà and Charlotte M. Verhagen for their contribution to the processing of the imaging data. We thank Olivia K.L. Hamilton, Irene M.C. Huenges Wajer, Bonnie Y.K. Lam, Adrian Wong and Xu Xin as members of the Meta VCI Map neuropsychology working group for their advice on neuropsychological data harmonization.

| Table S1. Selection of cognitive tests per cohort | | | | |
| --- | --- | --- | --- | --- |
| Cohort | **Attention & Executive functioning** | **Information processing speed** | **Language** | **Verbal memory** |
| Bundang VCI | 1. TMT B 2. Phonemic fluency | 1. TMT A 2. Digit Symbol Coding | 1. Boston Naming Test 2. Semantic fluency –animals | Seoul Verbal Learning Test:   1. Immediate recall 2. Delayed recall 3. Recognition |
| CASPER | 1. TMT B 2. Digit span forward 3. Digit span backward | 1. TMT A | 1. Semantic fluency – animals | Rey Auditory Verbal Learning Test   - 1. Immediate recall   2. Delayed recall   3. Recognition |
| COAST | 1. Digit span forward 2. Digit span backward | 1. Symbol Digit Modalities Test | 1. Boston Naming Test 2. Semantic fluency – animals | Word-List Recall   - 1. Immediate   2. Delayed   3. Recognition |
| CODECS | 1. TMT B 2. Phonemic fluency | 1. TMT A | 1. Semantic fluency –animals | N/A |
| DEDEMAS | 1. TMT B 2. Phonemic fluency | 1. TMT A 2. Digit Symbol Coding | 1. Boston Naming Test 2. Semantic fluency – animals | Word-List Memory Task   1. Immediate 2. Delayed 3. Recognition |
| Hallym VCI | 1. TMT B 2. Phonemic fluency | 1. TMT A 2. Digit Symbol Coding | 1. Boston Naming Test 2. Semantic fluency – animals | Seoul Verbal Learning Test   1. Immediate recall 2. Delayed recall 3. Recognition |
| PROCRAS | 1. TMT B 2. Phonemic fluency 3. Digit span forward 4. Digit span backward | 1. TMT A 2. Symbol Digit Modalities Test | 1. Boston naming Test 2. Semantic fluency – animals | Rey Auditory Verbal Learning Test   - 1. Immediate recall   2. Delayed recall |
| STROKDEM | 1. TMT B 2. Phonemic fluency | 1. TMT A 2. Digit Symbol Coding | 1. D080 – picture naming 2. Semantic fluency – animals | Free and Cued Selective Reminding Test   - 1. Immediate recall   2. Delayed free recall |
| USCOG | 1. Phonemic fluency 2. Digit span forward 3. Digit span backward | N/A | 1. Boston naming Test 2. Semantic fluency – animals | Rey Auditory Verbal Learning Test   - 1. Immediate recall   2. Delayed recall   3. Recognition |
| Abbreviations: N/A: not available, TMT: Trail Making Test. | | | | |

| Table S2. Cohort-specific baseline characteristics | | | | | | | | | | |
| --- | --- | --- | --- | --- | --- | --- | --- | --- | --- | --- |
| Cohort* | Bundang VCI  (n = 546) | CASPER  (n = 100) | COAST  (n = 2) | CODECS  (n = 11) | DEDEMAS  (n = 66) | Hallym VCI  (n = 534) | PROCRAS  (n = 161) | STROKDEM  (n = 135) | USCOG  (n = 13) | Total sample  (n = 1568) |
| Demographics and  clinical characteristics | | | | | | | | | | |
| Country of inclusion | South Korea | The Netherlands | Singapore | The Netherlands | Germany | South Korea | The Netherlands | France | The Netherlands | -- |
| Age in years, mean (SD) | 70.3 (10.7) | 64.2 (10.6) | 54.0 (9.9) | 57.4 (15.3) | 69.8 (8.6) | 65.1 (11.9) | 69.6 (9.5) | 64.9 (12.0) | 57.2 (17.7) | 67.3 (11.5) |
| Female, n (%) | 324 (59.3) | 26 (26.0) | 1 (50) | 5 (45.5) | 24 (36.4) | 232 (43.4) | 57 (35.4) | 83 (61.5) | 7 (53.8) | 626 (39.9) |
| Education level (STROKOG), n(%)† |  | | | | | | | | | |
| - - Lower than secondary school | 289 (52.9) | 41 (41.0) | 2 (100.0) | 3 (27.3) | 26 (39.4) | 292 (54.7) | 73 (45.3) | 85 (63.0) | 5 (38.5) | 816 (52.0) |
| - - Secondary school | 106 (19.4) | 18 (18.0) | 0 (0) | 2 (18.2) | 23 (34.8) | 126 (23.6) | 49 (30.4) | 15 (11.1) | 4 (30.8) | 343 (21.9) |
| - - Technical school or college | 26 (4.8) | 33 (33.0) | 0 (0) | 3 (27.3) | 16 (24.2) | 27 (5.1) | 34 (21.1) | 11 (8.1) | 3 (23.1) | 153 (9.8) |
| - - University or higher | 125 (22.9) | 8 (8.0) | 0 (0) | 3 (27.3) | 1 (1.5) | 89 (16.7) | 5 (3.1) | 24 (17.8) | 1 (7.7) | 256 (16.3) |
| NIHSS baseline, median (IQR) | 3 (2-5) | N/A | 9.5 (7-12) | 0 (0-2) | 2 (1-5)$ | 2 (1-4)* | 3 (2-4.5) | 0 (0-1)* | N/A | 2 (1-4)* |
| IQCODE, median (IQR) | 3.3 (3.1-3.7)* | 3.1 (3.0-3.3) | 3.1 (3.0-3.2) | N/A | N/A | 3.1 (3-3.3)$ | 3.0 (3.0-3.1) | 3.0 (3.0-3.1) | N/A | 3.1 (3-3.4)$ |
| Vascular risk factors, n (%) |  |  |  |  |  |  |  |  |  |  |
| History of stroke or TIA | 79 (14.5) | 6 (6.0) | 0 (0) | 0 (0) | 8 (12.1) | 74 (13.9)* | 33 (20.5) | 18 (13.3) | 2 (15.4)$ | 220 (14.0)* |
| Smoking present | 101 (18.5) | 17 (17.0) | 0 (0) | N/A | N/A | 148 (27.7)# | 35 (21.7) | 30 (22.2) | 3 (23.1)$ | 334 (21.3)# |
| Smoking past | 108 (19.8) | 58 (58.0) | 1 (50.0) | 3 (27.3) | N/A | 51 (9.6)# | 74 (46.0) | N/A | 1 (7.7)$ | 296 (18.9)$ |
| Hypercholesterolemia | 132 (24.2) | 85 (85.0%) | 1 (50.0) | 5 (45.5) | 22 (33.3) | 212 (39.7)# | 153 (95.0) | 59 (43.7) | 2 (15.4)$ | 671 (42.8)* |
| Hypertension | 420 (76.9) | 77 (77.0%) | 1 (50.0) | 6 (54.4) | 41 (62.1) | 329 (61.6)* | 117 (72.7) | 75 (55.6) | 3 (23.1)$ | 1069 (68.2)* |
| Diabetes mellitus | 174 (31.9) | 13 (13.0%) | 1 (50.0) | 2 (18.2) | 11 (16.7) | 157 (29.4)* | 45 (28.0) | 18 (13.3) | 1 (7.7)$ | 422 (26.9)* |
| Brain imaging | | | | | | | | | | |
| Imaging timing in days‡, median (IQR) | 5 (4-6) | 87 (81-99) | 3.5 (3-4) | 97 (52-183) | 4 (2.75-5) | 1 (1-2)* | 33 (27.5-40) | 3 (3-4) | 4 (3-6) | 4 (2-7)* |
| WMH volume in mL, median (IQR) | 14.6 (5.8-32.6) | 4.9 (1.9-11.4) | 0.1 (0.1-0.1) | 2.4 (1.3-11.2) | 2.8 (1.4-7.4) | 6.9 (2.7-18.3) | 4.8 (2.2-10.1) | 2.7 (1.2-4.8) | 3.8 (1.4-5.1) | 7.1 (2.8-19.2) |
| Acute infarct volume in mL, median (IQR)  Stroke subgroup, n | 3.4 (1.1-14.3) | 3.4 (0.9-13.2) | 76.3 (46.2-106.4) | 6.4 (1.1-21.8) | 2.1 (0.5-12.6) | 1.9 (0.9-8.3) | 4.4 (1.4-21.1) | 1.7 (0.6-8.7) | 19.0 (2.4-66.2) | 2.6 (1.0-12.3) |
| - - Small subcortical infarcts | 158 | 36 | 0 | 1 | 22 | 200 | 50 | 46 | 4 | 517 |
| - - Large infarcts | 283 | 50 | 2 | 0 | 33 | 219 | 86 | 78 | 8 | 759 |
| - - Infratentorial infarcts | 141 | 14 | 0 | 11 | 14 | 137 | 29 | 14 | 1 | 361 |
| Presence of old infarcts, n (%) | 101 (18.5) | 21 (21.0) | 1 (50.0) | 2 (18.2) | 10 (15.2) | 103 (19.3) | 27 (16.8) | 37 (27.4) | 0 (0) | 302 (19.3) |
| Presence of lacunes,  n (%) | 216 (39.6) | 30 (30.0) | 1 (50.0) | 5 (45.5) | 28 (42.4) | 181 (33.9) | 70 (45.5) | 38 (28.1) | 3 (23.1) | 572 (36.5) |
| Cognitive assessment | | | | | | | | | | |
| Cognitive assessment timing in days‡, median (IQR) | 103.5 (10-170) | 87 (81-99) | 106.8 (104-109.5) | 90 (90-90) | 187 (180-199) | 98 (90-104)* | 35 (28.5-40) | 189 (178-201) | 5 (3.5-12) | 98 (72.5-151)* |
| Attention and executive functioning, z-score, mean (SD) | -0.9 (1.1)$ | -0.7 (0.7) | -0.4 (1.8) | -1.0 (0.8) | -0.1 (0.9) | -0.7 (1.1)* | -0.7 (0.8)* | -0.8 (1.6)* | -0.8 (0.6) | -0.7 (1.0) |
| Information processing speed, z-score, mean (SD) | -0.9 (1.2)$ | -0.8 (1.3) | -2.6 (0.3) | -0.6 (0.7) | -0.1 (0.9) | -0.4 (1.1)* | -0.8 (1.1)* | -0.5 (1.1)* | N/A | -0.6 (1.2) |
| Language, z-score, mean (SD) | -1.1 (1.2)* | -0.4 (0.8)$ | -1.4 (1.5) | -0.6 (1.0) | 0.0 (0.8) | -0.5 (0.9) | -1.0 (0.9)* | -0.2 (0.7)* | -1.0 (1.3) | -0.7 (1.1) |
| Verbal memory, z-score, mean (SD) | -1.5 (1.1)* | 0.0 (1.0) | -1.6 (1.0) | N/A | 0.0 (0.8) | -0.7 (1.0) | -1.4 (0.9) | -0.2 (1.3)* | -1.4 (1.7)$ | -0.9 (1.2) |
| *Cohort specific in- and exclusion criteria are described in Weaver et al. (2021)^9^, supplementary material p.20 and Weaver et al. (2019)^12^, p.319.; †Education categories as defined by the STROKOG consortium^13^; ‡Days after index stroke; *Missing in <1%; *Missing in 1-10%; $Missing in >10% Abbreviations: SD, standard deviation; NIHSS, National Institute of Health Stroke Scale; IQR, interquartile range; TIA, transient ischemic attack; IQCODE, Informant Questionnaire for Cognitive Decline in the Elderly score; WMH, white matter hyperintensities.  This table was previously published in Stroke ^1^. | | | | | | | | | | |

**Table S3. Sensitivity analyses: mixed linear model analysis including 20 white matter tracts defined according to the JHU-atlas**

|  | | Attention & Executive functioning | | | Information processing speed | | | Language | | | Verbal Memory | | |
| --- | --- | --- | --- | --- | --- | --- | --- | --- | --- | --- | --- | --- | --- |
| Model | Independent variables | Coefficient | SE | P-value | Coefficient | SE | P-value | Coefficient | SE | P-value | Coefficient | SE | P-value |
| 1 | WMH volume | -0.010 | 0.001 | 5.528*^10-12^* | -0.011 | 0.002 | 1.323*^10-11^* | -0.009 | 0.001 | 9.237*10^-12^* | -0.003 | 0.001 | 0.017 |
| 2 | Model 1 + Anterior thalamic radiation L | -0.175 | 0.065 | 0.007 | -0.197 | 0.071 | 0.006 | -0.078 | 0.058 | 0.175 | -0.089 | 0.062 | 0.154 |
| 3 | Model 1 + Anterior thalamic radiation R | -0.089 | 0.071 | 0.212 | -0.117 | 0.077 | 0.13 | -0.096 | 0.067 | 0.149 | -0.076 | 0.071 | 0.282 |
| 4 | Model 1 + Corticospinal tract L | 0.102 | 0.126 | 0.381 | 0.045 | 0.128 | 0.722 | 0.062 | 0.103 | 0.550 | 0.137 | 0.116 | 0.237 |
| 5 | Model 1 + Corticospinal tract R | 0.171 | 0.115 | 0.139 | 0.209 | 0.125 | 0.096 | 0.001 | 0.107 | 0.992 | 0.060 | 0.113 | 0.598 |
| 6 | Model 1 + Cingulum (cingulate gyrus) L | 0.582 | 0.418 | 0.164 | 0.776 | 0.496 | 0.120 | -0.292 | 0.359 | 0.415 | -0.330 | 0.389 | 0.395 |
| 7 | Model 1 + Cingulum (cingulate gyrus) R | -1.209 | 0.685 | 0.078 | -1.575 | 0.794 | 0.047 | -1.478 | 0.570 | 0.010 | -0.789 | 0.608 | 0.194 |
| 8 | Model 1 + Cingulum (hippocampus) L | -0.042 | 7.824 | 0.992 | -1.360 | 7.800 | 0.855 | -1.552 | 6.814 | 0.818 | 2.445 | 7.225 | 0.736 |
| 9 | Model 1 + Cingulum (hippocampus) R | -5.831 | 6.231 | 0.347 | -2.622 | 6.755 | 0.692 | 1.268 | 5.975 | 0.834 | 6.826 | 6.359 | 0.283 |
| 10 | Model 1 + Forceps major | -0.076 | 0.051 | 0.131 | -0.132 | 0.054 | 0.014 | 0.010 | 0.046 | 0.837 | -0.049 | 0.050 | 0.327 |
| 11 | Model 1 + Forceps minor | 0.037 | 0.071 | 0.611 | -0.012 | 0.078 | 0.869 | -0.072 | 0.061 | 0.237 | -0.187 | 0.065 | 0.004 |
| 12 | Model 1 + Inferior fronto-occipital fasciculus L | -0.088 | 0.080 | 0.271 | 0.010 | 0.088 | 0.900 | 0.069 | 0.069 | 0.321 | -0.039 | 0.077 | 0.606 |
| 13 | Model 1 + Inferior fronto-occipital fasciculus R | -0.002 | 0.064 | 0.976 | 0.039 | 0.070 | 0.573 | 0.077 | 0.060 | 0.200 | -0.056 | 0.064 | 0.386 |
| 14 | Model 1 + Inferior longitudinal fasciculus L | -0.233 | 0.086 | 0.007 | -0.118 | 0.098 | 0.230 | -0.038 | 0.076 | 0.614 | -0.165 | 0.082 | 0.044 |
| 15 | Model 1 + Inferior longitudinal fasciculus R | -0.117 | 0.110 | 0.287 | -0.136 | 0.119 | 0.255 | -0.043 | 0.101 | 0.669 | -0.350 | 0.107 | 0.001* |
| 16 | Model 1 + Superior longitudinal fasciculus L | 0.087 | 0.043 | 0.043 | 0.123 | 0.047 | 0.009 | 0.047 | 0.038 | 0.216 | 0.050 | 0.042 | 0.242 |
| 17 | Model 1 + Superior longitudinal fasciculus R | 0.145 | 0.053 | 0.007 | 0.085 | 0.055 | 0.127 | 0.177 | 0.048 | <0.001* | 0.153 | 0.051 | 0.003 |
| 18 | Model 1 + Uncinate fasciculus L | 0.336 | 0.542 | 0.534 | 0.393 | 0.594 | 0.499 | 0.716 | 0.484 | 0.138 | 1.389 | 0.529 | 0.009 |
| 19 | Model 1 + Uncinate fasciculus R | 0.213 | 0.518 | 0.669 | -0.084 | 0.562 | 0.886 | 0.539 | 0.477 | 0.254 | 1.001 | 0.510 | 0.049 |
| 20 | Model 1 + Superior longitudinal fasciculus (temporal part) L | 0.075 | 2.302 | 0.973 | 4.818 | 2.506 | 0.054 | 4.554 | 1.994 | 0.022 | -1.723 | 2.208 | 0.434 |
| 21 | Model 1 + Superior longitudinal fasciculus (temporal part) R | 0.026 | 0.423 | 0.946 | 0.430 | 0.438 | 0.324 | 0.548 | 0.376 | 0.144 | -0.131 | 0.403 | 0.746 |

This table shows the results of the ROI-based analysis using mixed linear models including 20 white matter tracts defined according to the JHU atlas. Patients with an infarct in the white matter tract of interest were excluded from the analysis. The results are corrected for study site using random effects. A Bonferroni correction for 20 tests (i.e. 20 ROIs for major white matter tracts) was applied and a p-value <0.0025 was considered statistically significant (indicated by *). The independent variables (i.e. total WMH volume and regional WMH volumes) are not standardized. The coefficient therefore corresponds to the change in cognitive functioning (decrease or increase in z-score) associated with each 1 mL increase of the independent variables.

**Table S4. Mixed linear model analysis stratified according to stroke subtype**A. Cortical and large subcortical infarcts

|  | | Attention & Executive functioning | | | Information processing speed | | | Language | | | Verbal Memory | | |
| --- | --- | --- | --- | --- | --- | --- | --- | --- | --- | --- | --- | --- | --- |
| Model | Independent variables | Coefficient | SE | P-value | Coefficient | SE | P-value | Coefficient | SE | P-value | Coefficient | SE | P-value |
| 1 | WMH volume  +  Infarct volume | -0.012  -0.003 | 0.002  0.001 | 1.542*10^-7^*  <0.001* | -0.015  -0.006 | 0.003  0.001 | 1.666*10^-8^*  2.192*10^-8^* | -0.008  -0.005 | 0.002  0.001 | 0.001*  5.092*10^-8^* | -0.002  -0.003 | 0.002  0.001 | 0.299  <0.001* |
| 2 | Model 1 + Anterior thalamic radiation L | -0.222 | 0.102 | 0.030* | -0.287 | 0.112 | 0.010* | -0.103 | 0.093 | 0.265 | -0.218 | 0.099 | 0.027* |
| 3 | Model 1 + Anterior thalamic radiation R | -0.124 | 0.109 | 0.248 | -0.133 | 0.118 | 0.254 | -0.043 | 0.102 | 0.666 | -0.037 | 0.106 | 0.720 |
| 4 | Model 1 + Forceps major | -0.001 | 0.084 | 0.950 | -0.233 | 0.085 | 0.006* | 0.088 | 0.076 | 0.251 | 0.061 | 0.079 | 0.444 |
| 5 | Model 1 + Inferior fronto-occipital fasciculus L | -0.119 | 0.131 | 0.353 | -0.127 | 0.143 | 0.377 | 0.087 | 0.111 | 0.435 | -0.005 | 0.123 | 0.964 |

B. Small subcortical infarcts

|  | | Attention & Executive functioning | | | Information processing speed | | | Language | | | Verbal Memory | | |
| --- | --- | --- | --- | --- | --- | --- | --- | --- | --- | --- | --- | --- | --- |
| Model | Independent variables | Coefficient | SE | P-value | Coefficient | SE | P-value | Coefficient | SE | P-value | Coefficient | SE | P-value |
| 1 | WMH volume | -0.010 | 0.002 | 6.653*10^-6^* | -0.011 | 0.003 | 3*10^-5^* | -0.013 | 0.002 | 1.12*10^-10^* | -0.009 | 0.002 | 0.0001* |
| 2 | Model 1 + Anterior thalamic radiation L | -0.218 | 0.109 | 0.054 | -0.288 | 0.121 | 0.018* | -0.270 | 0.094 | 0.004* | -0.029 | 0.107 | 0.767 |
| 3 | Model 1 + Anterior thalamic radiation R | -0.095 | 0.117 | 0.444 | -0.141 | 0.131 | 0.288 | -0.276 | 0.107 | 0.010* | -0.081 | 0.118 | 0.479 |
| 4 | Model 1 + Forceps major | -0.076 | 0.086 | 0.356 | -0.005 | 0.096 | 0.938 | 0.056 | 0.077 | 0.477 | -0.082 | 0.086 | 0.337 |
| 5 | Model 1 + Inferior fronto-occipital fasciculus L | -0.137 | 0.120 | 0.274 | 0.023 | 0.137 | 0.844 | -0.054 | 0.107 | 0.613 | -0.057 | 0.120 | 0.629 |

C. Infratentorial infarcts

|  | | Attention & Executive functioning | | | Information processing speed | | | Language | | | Verbal Memory | | |
| --- | --- | --- | --- | --- | --- | --- | --- | --- | --- | --- | --- | --- | --- |
| Model | Independent variables | Coefficient | SE | P-value | Coefficient | SE | P-value | Coefficient | SE | P-value | Coefficient | SE | P-value |
| 1 | WMH volume | -0.012 | 0.003 | 2.012*10^-5^* | -0.009 | 0.003 | 0.002* | -0.010 | 0.003 | 0.0001* | 0.001 | 0.003 | 0.861 |
| 2 | Model 1 + Anterior thalamic radiation L | -0.14 | 0.114 | 0.222 | -0.155 | 0.121 | 0.198 | 0.057 | 0.104 | 0.593 | -0.061 | 0.111 | 0.574 |
| 3 | Model 1 + Anterior thalamic radiation R | 0.009 | 0.133 | 0.907 | -0.016 | 0.133 | 0.901 | 0.043 | 0.124 | 0.731 | -0.126 | 0.128 | 0.322 |
| 4 | Model 1 + Forceps major | -0.272 | 0.085 | 0.001* | -0.175 | 0.092 | 0.053 | -0.123 | 0.078 | 0.110 | -0.093 | 0.084 | 0.265 |
| 5 | Model 1 + Inferior fronto-occipital fasciculus L | 0.011 | 0.168 | 0.875 | 0.202 | 0.170 | 0.229 | 0.244 | 0.147 | 0.096 | -0.020 | 0.161 | 0.891 |

This table shows the ROI-based analysis using a mixed linear model including 4 significant white matter tracts stratified according to infarct subtype. Patients with an infarct in the white matter tract of interest were excluded from the analysis. The results are corrected for study site using random effects. A p-value <0.05 was considered statistically significant (indicated by *). The independent variables (i.e. total WMH volume and regional WMH volumes) are not standardized. The coefficient therefore corresponds to the change in cognitive functioning (decrease or increase in z-score) associated with each 1 mL increase of the independent variables.

**References**

1. de Kort FAS, Coenen M, Weaver NA, et al. White Matter Hyperintensity Volume and Poststroke Cognition: An Individual Patient Data Pooled Analysis of 9 Ischemic Stroke Cohort Studies. *Stroke*. October 2023. doi:10.1161/STROKEAHA.123.044297

2. Kuijf HJ, Casamitjana A, Collins DL, et al. Standardized Assessment of Automatic Segmentation of White Matter Hyperintensities and Results of the WMH Segmentation Challenge. *IEEE Trans Med Imaging*. 2019;38(11):2556-2568. doi:10.1109/TMI.2019.2905770

3. Camarasa R, Doué C, de Bruijne M, Dubost F. Segmentation of White Matter Hyperintensities with an Ensemble of Multi-Dimensional Convolutional Gated Recurrent Units. [online] available from: https://wmh.isi.uu.nl/wp-content/uploads/2018/08/coroflo.pdf.

4. Chen S, Sedghi Gamechi Z, Dubost F, van Tulder G, de Bruijne M. An end-to-end approach to segmentation in medical images with CNN and posterior-CRF. *Med Image Anal*. 2022;76:102311. doi:10.1016/j.media.2021.102311

5. Douven E, Schievink SHJ, Verhey FRJ, et al. The Cognition and Affect after Stroke - a Prospective Evaluation of Risks (CASPER) study: rationale and design. *BMC Neurol*. 2016;16(1):65. doi:10.1186/s12883-016-0588-1

6. Biesbroek JM, Kuijf HJ, Weaver NA, Zhao L, Duering M, Biessels GJ. Brain infarct segmentation and registration on MRI or CT for lesion-symptom mapping. *Journal of Visualized Experiments*. 2019;2019(151). doi:10.3791/59653

7. Fonov V, Evans AC, Botteron K, Almli CR, McKinstry RC, Collins DL. Unbiased average age-appropriate atlases for pediatric studies. *Neuroimage*. 2011;54(1):313-327. doi:10.1016/j.neuroimage.2010.07.033

8. Duering M, Biessels GJ, Brodtmann A, et al. Neuroimaging standards for research into small vessel disease—advances since 2013. *Lancet Neurol*. 2023;22(7):602-618. doi:10.1016/S1474-4422(23)00131-X

9. Weaver NA, Kuijf HJ, Aben HP, et al. Strategic infarct locations for post-stroke cognitive impairment: a pooled analysis of individual patient data from 12 acute ischaemic stroke cohorts. *Lancet Neurol*. 2021;20(6):448-459. doi:10.1016/S1474-4422(21)00060-0

10. Berres M, Monsch AU, Bernasconi F, Thalmann B, Stähelin HB. Normal ranges of neuropsychological tests for the diagnosis of Alzheimer’s disease. *Stud Health Technol Inform*. 2000;77:195-199.

11. Ryan J, Lopez S. *Wechsler Adult Intelligence Scale-III. In: Understanding Psychological Assessment*. Boston, MA: Springer US; 2001.

12. Weaver NA, Zhao L, Biesbroek JM, et al. The Meta VCI Map consortium for meta-analyses on strategic lesion locations for vascular cognitive impairment using lesion-symptom mapping: Design and multicenter pilot study. *Alzheimer’s and Dementia: Diagnosis, Assessment and Disease Monitoring*. 2019;11:310-326. doi:10.1016/j.dadm.2019.02.007

13. Lo JW, Crawford JD, Desmond DW, et al. Profile of and risk factors for poststroke cognitive impairment in diverse ethnoregional groups. *Neurology*. 2019;93(24). doi:10.1212/WNL.0000000000008612
